# Effectiveness of a short-course in improving knowledge and skills on evidence-based practice

**DOI:** 10.1186/1471-2296-12-64

**Published:** 2011-06-30

**Authors:** Josep M Argimon-Pallàs, Gemma Flores-Mateo, Josep Jiménez-Villa, Enriqueta Pujol-Ribera

**Affiliations:** 1 Divisió d'Avaluació, Servei Català de la Salut, Barcelona, Spain; 2 Institut d'Investigació en Atenció Primària Jordi Gol, Barcelona, Spain

**Keywords:** Evidence Based Practice, effectiveness, course

## Abstract

**Background:**

To assess the effectiveness (change in knowledge and skills measured by the Fresno test) of a short course in Evidence Based Practice (EBP) carried out in a group of family medicine residents

**Methods:**

Before-after study. Participants' were 152 Family Medicine residents in their second year of the training programme. Settings were Primary Care Teaching Units in Catalonia. Intervention was comprised of a four half-day training course designed to develop the knowledge and skills required to practice evidence-based care. The main outcome measure was change in EBP knowledge and skills, measured using the Spanish version of the Fresno test (score range, 0-212)

**Results:**

The mean difference between pre-test and post-test was 47.7, a statistically significant result with 95% CI of 42.8-52.5 (*p *< 0.0001). An important improvement was observed in the questions related to calculations such as sensitivity, specificity, the absolute risk reduction or the number needed to treat. A more modest increase was found in the residents' knowledge and skills in finding the best clinical evidence, and appraising the validity and applicability of an article. Finally, a weak and non-statistically significant improvement was found in formulating a clinical question.

**Conclusions:**

The study provides evidence for responsiveness to changes in knowledge and skills in EBP after an educational intervention.

## Background

Several systematic reviews have consistently reported that Evidence Based Practice (EBP) training results in improvements in participants' knowledge of methodological and statistical issues and enhances their attitudes towards the use of medical literature in clinical decision making [1-4]. Nevertheless, these findings need to be interpreted with considerable caution since most of the studies had poor internal validity [5]. One of the criticisms is that studies give few details about how effectiveness is measured. If a questionnaire was used the authors provided little detail on how the questionnaires were developed and validated, how they were administrated and how long before the intervention [5].

Shaneyfelt et al. [6] reviewed the available EBP teaching instruments methods and identified high-quality instruments for evaluating the EBP competence of individual trainees, determining the effectiveness of EBP curricula, and assessing EBP behaviours with objective outcome measures. Within high-quality instruments the Fresno Test [7] evaluates the most of EBP steps and measures change in knowledge and skills. It begins with the presentation of two scenarios that suggest clinical uncertainty. Short answer questions about the clinical scenarios require the candidate to formulate a focused question, identify the most appropriate research design for answering the question, show knowledge of electronic database searching, identify issues important for determining the relevance and validity of a given research article, and discuss the magnitude and importance of research findings. Unlike multiple choice or true-false questions, the open ended questions require examinees to show higher order thinking in response to an authentic task.

However, Ramos et al [7] did not perform an assessment of the responsiveness of the test. Responsiveness is the extent to which instruments are sensitive enough to detect the smallest difference considered clinically relevant [8]. Recently the test has been translated and validated into Spanish, including the assessment of the responsiveness [9]. The objective of this study is to show the effectiveness (change in knowledge and skills measured by the Fresno test) of a short course in EBP carried out in a group of family medicine residents, which allowed the assessment of the responsiveness of the test.

## Methods

### Study design

Before and after study

### Participants, setting and context

Medical residents in their second year of the Family Medicine training programme in Catalonia, before they have had any formal training in EBP during their residence program. The setting of the study was the Primary Care Teaching Units in Catalonia (PCTU). At the beginning of the Family Medicine residence program (which lasts four years) the medical residents are enrolled into a PCTU. Each PCTU comprises several health centres in a defined geographical area. There are seventeen PCTU in Catalonia.

The number of medical residents in each PCTU ranged from 2 to 52 in 2007. Residents are not exposed to EBP formal training until the second year of their program. In the year 2007 there were 202 residents in their second year of training.

The following variables were recorded for each resident: age, sex, year of graduation in medicine, courses in EBP completed prior to the educational intervention and time required in filling-in the test.

### Educational intervention

The educational intervention is an intensive and interactive four half-day sessions designed to develop the knowledge and skills required to practice evidence-based care. The course is compulsory for the residents in this specialty. At the end of the instruction an evaluation is mandatory. Until now the assessment had been a multiple choice questionnaire.

The course was modelled after the steps of EBP first described in 1992 by Cook et al [10]. Sessions featured a mix of interactive lectures, workshops and case-based studies around six topics: (a) writing a clinical question; (b) searching the medical literature; (c) selecting and obtaining the evidence; (d) critical appraisal of systematic reviews, randomised clinical trials and diagnostic test; (e)Interpreting the clinical relevance and precision of the results; and (f) application of evidence to clinical care.

In the first session, the residents were introduced to the concept of EBP and how it is used in clinical practice. They were taught to recognize situations that involve clinical uncertainty and whether their information needs constitute a background or foreground question. The students learned that EBP skills are helpful in answering foreground questions, including questions about therapy, diagnosis, prognosis, or harm. As a method of teaching lecturers presented clinical scenarios and asked the participants to frame a focussed, answerable question in a structured four part format (patient-intervention/exposure-comparator-outcome) that could lead to effective search and appraisal strategies. The Fresno Test evaluates these skills by presenting two clinical scenarios and asking the student to form two focussed, structured and answerable questions. In addition, this session covered levels of evidence. The Fresno Test asks the participant to select the best design for answering the formulated question.

The second session was dedicated to developing searching skills and improving searching efficiency. Theoretical instruction backed by a supervised practical session with online connection was used as a method of teaching. A variety of databases was taught such as Cochrane, MEDLINE, CINAHL, SumSearch, Tripdatabase.com with the relative benefits discussed. The Fresno Test asks the students to name possible resources of information where clinicians can go to find an answer to questions like these defined previously. The participant is also asked to name one characteristic of each resource that makes it useful, in order to show that he or she understands the strengths and weaknesses of the different sources of evidence. Finally, the participant should write out an effective and comprehensive search strategy in PubMed for one of these questions.

The third session reviewed critical appraisal of evidence for validity, clinical relevance, and applicability. The student was taught to appraise the validity of a randomised clinical trial. The appraisal included: the suitability of the type of study to the type of question asked, the design of the study and sources of bias, the reliability and validity of outcome measures chosen, and the suitability and robustness of the analysis employed. The student appraised the importance of the outcomes and translated them into clinically meaningful summary statistics, such as number needed to treat, absolute risk reduction and relative risk reduction, and interpretation of confidence intervals. Most of the questions of the Fresno Test are related to the critical appraisal skills.

In the last session, participants were asked to select a patient, formulate a focused clinical question on diagnosis, search the evidence, and appraise an article with respect to validity and applicability to their patient. Instructors reviewed measures of diagnosis including: sensitivity, specificity, positive and negative predictive value, and likelihood ratios. One question of the Fresno Test is devoted to the calculation of these indices. In the second part of the session students were taught to explore the generalisability of the evidence to the specific scenario, and 'particularising' outcomes by adjusting for patient-specific risks. The Fresno test asks a particular question about generalisability.

Time was set aside after each session for the group to reflect, consider how they would apply the new skills and knowledge in their daily practice. It was also an opportunity to ask questions and review.

The lecturers were the epidemiologists working in each PCTU. The lecturer conducted the module with the assistance of a health librarian during the session of searching the literature. Regarding the present study, lecturers were advised not to modify their sessions with a view to coaching for the test.

### Outcome measure

To allow the assessment of the effectiveness of the educational intervention the Fresno test was administered before and after the educational intervention.

Repeated administration of measures, particularly those focussing on knowledge, may over-estimate the educational effect and therefore the responsiveness of the test. To minimise the recall bias two sets of the test with different scenarios and calculation questions were prepared. Moreover the pre-test was administered four weeks before starting the educational module.

Residents were invited four weeks before starting the educational module to attend a conference about research in primary care and the importance of applying the results of this research into clinical practice. The project of the Fresno Test was also presented. The sessions were decentralised by Health Regions. At the end of each session participants received the test (before test). The post-test was administered on the last day of the course (after test). Educational activities were initiated in October 2007 and were completed in June 2008.

The complete process of scoring the test, as presented by Ramos et al [7], can be found in the BMJ website (http://www.bmj.com/cgi/content/full/326/7384/319/DC1). Two investigators (JA and GF-M) scored the short answer questions using the standardised grading rubrics. Inter-rater reliability was assessed using intra-class correlation coefficient (ICC) for the overall score. For each question, the rubric specifies explicit grading criteria. For instance, the first item asks the respondent to write a focused clinical question. Responses are scored based on their inclusion of a patient population, an intervention, a comparison, and an outcome. They used four or five grading categories (not evident, minimal and/or limited, strong, excellent), each of which is associated with a point value. For instance, no mention of a patient population earns 0 points (not evident), the use of a general patient identifier is a limited answer (2 points), mentioning a single specific patient descriptor is a strong answer (4 points), and using numerous relevant descriptors is excellent (6 points). Each criterion is scored into these categories. The sum of points for all criteria is the score for that item (Table 1).

**Table 1:** Sample rubric: formulating a Clinical Question

	Patient	Intervention/Exposure	Comparison	Outcome
Excellent (3 points)	> 1 appropriate descriptor	Specific Intervention	Specific intervention	Objective, patient-oriented

Strong (2 points)	1 appropriate descriptor	Type of intervention	Type of intervention	Surrogate marker

Limited (1 point)	Descriptor lacking specificity	Intervention	Comparison	Non-specific outcome

Not evident (0 points)	None of above	None of above	None of above	None of above

for the item described above, limited performance in each category would result in a score of 8. They therefore considered any total less than 8 for a question as "not evident." A score of 8-15 was defined as a limited response, 16-23 as a strong response, and 24 as an excellent response. Different questions have a different point value for each grading category. By this process, each short answer response is assigned a numerical score (from 0 to 24 points, and some questions only 4 points) and designated pass or fail. The total test score is the sum of points for all items. The maximum possible score is 212 points.

The authors of the original test assigned a cut off for a "passing" answer. They used their professional judgment of adequate mastery of the material to set this cut off as the midpoint of the strong category of response.

### Sample size

The sample size was based upon the ability to detect a minimal important difference before and after of 10 points on the test (standard deviation = 20) with 90% statistical power and two side alpha level of 0.05. Given these assumptions we would need 70 participants. Assuming that 20% of those completing the questionnaire had incomplete or invalid data, the sample was extended to 85 subjects.

### Statistical analysis

Descriptive statistics were used to summarize demographic characteristics and scores on items of the test. Categorical variables were summarized in terms of frequencies and percentages, and for each continuous variable, the mean, standard deviation are presented. The change in knowledge and skills was computed as the difference between scores post-course and scores pre-course and tested using a paired t-test. Thus a positive difference meant a gain in knowledge and skills. We computed the proportion of gain in knowledge and skills using the maximal points that can be achieved (212) as denominator. Since those residents with lower pre-test scores have more scope for improvement we also measured a relative change in score adjusted for differences in score before the course. This score was calculated as a ratio, with the numerator being the difference between the post-test and the pre-test scores and the denominator being the maximum obtainable value minus the pre-test score [11]. Chi squared test was used to analyse the proportion that attained the pass mark.

Responsiveness statistics used in the analysis were the effect size (ES) and the standardised response mean (SMR). The ES was calculated as the difference between the mean baseline and follow-up scores on the measure, divided by the standard deviation of the baseline scores. The SRM was calculated as the mean change in scores divided by the standard deviation of these changes [12].

Several cases were excluded of the analysis: the resident who did not fill-in the pre-test and those who failed to answer the post-test were not taken into account for the responsiveness analysis, as well as those residents who did not attend at least three half-days of the EBP course.

The questionnaires were completed on paper and all data from raters were entered electronically at the end of the course. Statistical analyses were conducted by using Stata software version 9.0 (STATA Corp, College Station, TX) and with SPSS software version 15.0 for Windows (SPSS, Chicago, IL, USA).

### Ethical approval

The study was approved by the Ethical Committee at IDIAP Jordi Gol i Gurina

## Results

196 of 202 residents in their second year of training took part in the study. 190 (94.1%) residents completed the pre-test, and 158 (78.2%) residents returned the post-questionnaire. Overall, 152 residents (75.2%) returned both sets of the questionnaire. The main reasons for partial completion were failure to submit the questionnaire after the course (n = 17), failure to participate in the course (n = 15), failure to submit the pre-test (n = 6), and failure of identification (n = 6).

The average age of the participants was 31 years (SD = 8.0) and 76.3% were women. No differences in age or sex were observed between the group of residents that completed only the pre-test questionnaire and the group of residents who returned both sets of questionnaires. However, the residents that did not return the post-test questionnaire had on average lower baseline scores in the pre-test than the group of residents who returned both sets of questionnaires (54.8 vs 63.9; p = 0.03).

Only 44 residents (23.2%) stated that they had formal, structured training in evidence based practice prior to the course. Residents who self-reported former training had higher scores in the pre-test than the rest of the residents [75.9 (SD = 29.2) vs 57.6 (SD = 23.1): CI 95%: 9.1-25.6; p < 0.0001].

The overall inter-rater reliability was 0.95 and 0.85 in the pre-test and post-test questionnaire, respectively

On the pre-test survey the average score for residents was 63.9 (SD = 24.3). On the post-test survey, the average score was 111.6 (SD = 30.4). Using a difference score as the criterion in a paired *t*-test, the mean difference between pre-test and post-test was 47.7, a statistically significant result with 95% CI of 42.8-52.5 (*p *< 0.0001). In percentage, the residents gained on average 22.5% of score of the total possible score. When adjusted for the individual potential for improvement, the residents gained on average 28.8%.

In the subgroup of residents with former training in EBP the difference before and after the intervention was: 45.8 (CI 95%: 35.5-56.2; p < 0.0001)

The observed ES for the residents was 1.77 (CI 95%: 1.57-1.95), and the SMR was 1.65 (CI 95%:1.47-1.82). In the subgroup of residents with former training the responsiveness indices were similar to those found in the whole group of residents: ES 1.78 (CI 95%: 1.37-2.17); SMR was 1.60 (CI 95%:1.24-1.96)

Table 2 shows the percentage of residents with a passing score in each question before and after the educational intervention. An important improvement was observed in the questions related to calculations such as sensitivity, specificity, the absolute risk reduction or the number needed to treat. A more modest increase was found in the residents' knowledge and skills in finding the best clinical evidence, and appraising the validity and applicability of an article. Finally, a weak and non-statistically significant improvement was found in formulating a clinical question.

**Table 2:** Percentage of residents with a passing score before and after the educational intervention and 95% confidence interval of the difference

	Before	After	CI 95%
Q1 Formulate Question	22.9	28.3	-2.1-13.1
Q2 Sources of information	12.3	22.3	3.3-16.1
Q3 Search	04.8	22.6	12.0-23.6
Q4 Study Design	22.9	37.7	6.7-22.5
Q5 Relevance	14.4	23.9	2.7-16.3
Q6 Internal validity	28.2	38.2	2.1-18.4
Q7 Magnitude of effect	1.6	14.5	8.4-17.7
Q8 Sensitivity	42.2	82.3	32.2-47.6
Specificity	34.0	78.5	36.9-52.4
Positive Predictive Value	32.6	81.5	41.5-56.5
Negative Predictive Value	25.9	79.9	46.4-61.1
Positive Likelihood Ratio	8.2	47.8	32.5-46.6
Q9 Absolute Risk Reduction	21.3	81.0	52.7-66.7
Relative Risk Reduction	06.5	63.9	52.1-64.3
Number Needed to Treat	10.6	60.3	42.7-56.9
Q10 Confidence Interval	13.2	48.3	27.7-42.6
Q11 Best Study Design, Diagnosis	24.0	44.0	11.7-27.8
Q12 Best Study Design, Prognosis	24.9	44.9	12.0-28.3

## Discussion

This study shows that a structured short course in EBP produces educationally gains in EBP knowledge and skills measured using a validated tool.

The results of the present study are consistent with other studies that have demonstrated that core EBP can be taught effectively to health care workers. The effect size in these studies varies according the length of the workshop or course. When the duration and frequency was less than half-day the effect size ranged from 0.36 (IC 95%:0.04-0.69) [13] to 0.41 (IC95%: 0.22-0.61) [14]. Taylor et al [15] found an effect size of 0.31(IC 95%:0.27-0.36) after 10 one-hour workshops (one workshop per week). In contrast, in a more similar study, Fristche et al [16] observed an effect size of 1.32 (IC95%: 1.11-1.53) after a structured three-day course.

The studies for which an effect size could not be calculated show similar results. Linzer at al [11] demonstrated in a randomised controlled trial that knowledge increased more in the group of participants who attended a journal club and a trend was found toward more knowledge gained as more sessions were attended. Ability to appraise critically a test article increased slightly, but there was no statistically significant difference between groups. Green et al. [17] used an EBP curriculum based on adult learning theory to show that a 7-week EBP curriculum improved skills of Internal Medicine Residents. Smith et al. [18] demonstrated a similar result after a 14-hour intervention for all core EBP skills except critical appraisal. Dorsh et al. [19] assessed the impact of an evidence-based medicine course on students' self-perception of EBP skills. A statistically significant increase was found in the students' self-assessment of skills. Students reported using the journal literature significantly more frequently than before, although textbooks remained their number one resource. Statistically significant improvement in student performance was also found on the post-test, although the level of improvement was more modest than that found on the post-surveys.

Two articles deserve an in depth commentary since they used the Fresno Test as the instrument for measuring changes in knowledge and skills [20,21]. However, their results are not strictly comparable because the authors adapted the test and the scoring system to match their objectives and their teaching modules. Dinkevich et al. [21] demonstrated the effectiveness of a brief teaching module developed to improve EBP addressed to second and third year paediatric residents. They used nine questions from the Fresno Test to evaluate four core EBP skills. The questions on articles about diagnosis and prevention were not used because those skills were not taught in their training module. The ability to formulate clinical questions was evaluated only by one question. The grading system of the Fresno Test was adapted too. Answers were assigned a score of 0 (inadequate skill) or 1 (adequate skill). Post intervention, the mean score increased to 63% with improvement in each EBP category. A mean of 4.08 more questions (out of 9) were answered correctly after the training (95% CI of 3.44-4.72).

McCluskey [20] recruited 114 self-selected occupational therapists. The intervention included a 2-day workshop combined with outreach support for eight months. Support involved e-mail and telephone contact and a workplace visit. Measures were collected at baseline, post-workshop, and eight months later. The primary outcome was knowledge, measured using an adapted Fresno Test (total score 0 to 156). Five of the 12 more advanced statistical questions were removed (for example, those about sensitivity, specificity, and number needed to treat), since these were not taught in the workshop curriculum. Three sets of different clinical scenarios were written for each test administration (i.e. baseline, post-workshop and follow-up). Post-workshop, there were significant gains in knowledge which were maintained at follow-up. The mean difference in the Adapted Fresno Test total score was 20.6 points (95% CI, 15.6 to 25.5). The change from post-workshop to follow-up was small and non-significant (mean difference 1.2 points, 95% CI, -6.0 to 8.5). The effect size for knowledge outcome was 0.91 (CI 95%: 0.64-1.17) lower than the observed in our study.

Although some of these factors may have introduced bias, the observed effect was large enough and it is unlikely that it could be totally explained solely by those potential biases. Furthermore, this study was performed in routine conditions reflecting real situations, with multiple lecturers who were advised not to modify their sessions with a view to coaching for the test. When several lecturers are involved there is always some difficulty in standardising the intervention. Lack of standardisation inflates error variance and decrease the chance of obtaining true differences [22].

In the present study, Family Medicine residents improved in all domains of EBP except for formulating a question. Several factors may explain this result. Firstly, it is possible that lecturers did not pay the same attention to this step compared to other domains. Nevertheless, this is unlikely since lecturers were experienced on EBP teaching and formulating a well-focused question is the first and arguably the most important step in the EBP process. In this study, lecturers used a specialized framework, called PICO (Patient problem, Intervention, Comparison, and Outcome) [23], to form the question and facilitate the literature search. In some places the PICO framework was expanded to PICOTT, adding information about the type of question being asked (therapy, diagnosis, prognosis, harm, etc.) and the best type of study design for that particular question. Using this framework helps the clinician articulate the important parts of the clinical question most applicable to the patient and facilitates the searching process by identifying the key concepts for an effective search strategy [24]. Secondly, to gain knowledge and skills in this area it is necessary to practice frequently with different scenarios. This is difficult to do in a teaching module where many topics have to be explained in a short period of time. In a near future may be worthwhile to reconsider the time allocated to each step of the EBP process in the teaching module. Thirdly, we provided residents with clinical scenarios which did not convey directly to a well-focused question. If we had provided, for instance, a clinical scenario where a new treatment was compared to a placebo, it might have been unrealistic in their simplicity and transfer to the PICO template. In the clinical setting the work of generating questions from clinical situations and translating them into the PICO format is usually more difficult. Family residents showed only a modest increase in searching and appraisal skills. It seems to be easier to gain knowledge on how to calculate clinically meaningful summary statistics than to acquire skills because they require more practice. In a near future it may be worthwhile to reconsider the time allocated to each step of the EBP process in the teaching module.

Evaluation of educational interventions should not only be concerned about a gain in knowledge and skills, but also on how this gain is transferred to workplace (behavioural or attitudes change) and impact on patients (health outcomes). However, the Fresno Test was not developed for measuring behavioural or attitudes change, and the present study was not designed either for assessing even short term behavioural or attitudes change.

Factors other than the course could be partially responsible for the observed effect. The interval before and after which the test is to be given is a relevant decision; a too short interval may over-estimate changes in knowledge because of recall bias. At the design phase, it was decided that the time elapsed between the pre and post-questionnaire administration should be four weeks, and to implement measures to prevent recall bias. The most important measure was to administer after the intervention new clinical vignettes and a new set of numeric examples for calculation questions. We did not change the order of the questions because it might alter the results, and it was felt that this risk was not worthwhile in order to avoid recall bias. On the other hand, the inability to blind for intervention could have led to improvement due to awareness of being evaluated (Hawthorne effect), or for studying at home in advance of the course [5]. Since we used a before-after design we could not control for a potential Hawthorne effect. Furthermore, the inability to blind the assessment (scorers knew if the test being evaluated was administered before or after the intervention) could also have biased the results. Non-respondents can be less knowledgeable, less confident and less engaged than respondents leading to an over-estimation of the truly effect. When considering evidence of responsiveness in this study, it is important to take into account that the baseline scores of knowledge and skills were quite low giving more scope for the instrument to be sensitive to change. As a matter of fact, McCluskey and Bishop [20] have demonstrated, using an adaptation of the Fresno Test, that the test is most useful for evaluating change in novice learners. Several other factors may influence the responsiveness of a measure, including, but not limited to, the content of the measure, the construct validity of the measure, the error associated with the scores, the existence of floor or ceiling effects and the criterion used to identify subjects as changed or not changed.

## Conclusions

The results of the present study have several implications. They provide evidence for responsiveness to changes in knowledge and skills in EBP after an educational intervention, and therefore the test can be used as an outcome measure in randomized clinical trials. The results also indicate that building up a well focussed clinical question was more challenging than any other EBP' domain. In the near future much more attention should be paid on how to build up a clinical question. Without a well-focused question, it can be very difficult and time consuming to identify appropriate resources and search for relevant evidence.

## Competing interests

The authors declare that they have no competing interests.

## Authors' contributions

JMA, GFM and JJV contributed to its design. JMA and GFM analysed & interpreted the data. JMA, GFM and EPR drafted the manuscript and all authors contributed to its revision. All authors approved the final version of the article to be published.

## Pre-publication history

The pre-publication history for this paper can be accessed here:

http://www.biomedcentral.com/1471-2296/12/64/prepub
